# Impact of school-based malaria intervention on primary school teachers’ time in Malawi: evidence from a time and motion study

**DOI:** 10.1186/s12936-022-04324-1

**Published:** 2022-10-27

**Authors:** Jobiba Chinkhumba, Victor Kadzinje, Gomezgani Jenda, Michael Kayange, Don P. Mathanga

**Affiliations:** 1 School of Public Health and Family Medicine, Department of Health Systems and Policy, Kamuzu University of Health Sciences, Blantyre, Malawi; 2 Health Economics and Policy Unit, Kamuzu University of Health Sciences, Blantyre, Malawi; 3 Save the Children International, Malawi Office, Lilongwe, Malawi; 4 Ministry of Health, National Malaria Control Programme, Lilongwe, Malawi; 5 School of Public Health and Family Medicine, Department of Community Health, Kamuzu University of Health Sciences, Blantyre, Malawi

**Keywords:** School-based health, Time and motion study, Learner Treatment Kit, Malaria

## Abstract

**Background:**

School-based health (SBH) programmes that are contingent on primary school teachers are options to increase access to malaria treatment among learners. However, perceptions that provision of healthcare by teachers may be detrimental to teaching activities can undermine efforts to scale up school-based malaria control. The objective of this study was to assess the impact of school-based malaria diagnosis and treatment using the Learner Treatment Kit (LTK) on teachers’ time.

**Methods:**

A time and motion study was conducted in 10 primary schools in rural Malawi. Teachers who had been trained to diagnose and treat uncomplicated malaria were continuously observed in real time during school sessions and the time they spent on all activities were recorded by independent observers before and after LTK implementation. A structured form, programmed digitally, was used for data collection. Paired sample t-tests were used to assess pre-post differences in average hours teachers spent on the following key activities: direct teaching; indirect teaching; administration; LTK and non-teaching tasks. Multivariable repeated measures mixed regression models were used to ascertain impact of LTK on average durations teachers spent on the key activities.

**Results:**

Seventy-four teachers, trained to use LTK, were observed. Their mean age and years of teaching experience were 34.7 and 8.7, respectively. Overall, 739.8 h of teacher observations took place. The average time teachers spent in school before relative to after LTK was 5.8 vs. 4.8 h, p = 0.01. The cumulative percentage of time teachers spent on core teaching activities (teaching and administration) was approximately 76% and did not change substantially before and after LTK. Some 24.3% of teachers’ time is spent on non-teaching activities. On average, teachers spent 2.9% of their time providing LTK services daily. Per day, each teacher spent less time on administrative (0.74 vs. 1.07 h, p = 0.02) and non-teaching activities (0.96 vs. 1.41 h, p = 0.01) during LTK compared with the period before LTK.

**Conclusion:**

School-based health (SBH) programmes are not detrimental to teaching activities. Teachers manage their time to ensure additional time required for SBH services is not at the expense of teaching duties. Programming and policy implications of tasking teachers with SBH does not have substantial opportunity costs. Teachers should continue delivering SBH programmes to promote learners’ health.

## Background

School-based health (SBH) programmes have gained traction over the past decades based on the recognition that ill-health can have significant negative impact on educational outcomes [1, 2]. As a result, several countries in the sub-Saharan region have taken the strategic decision to use primary schools as platforms for delivering health and nutrition interventions, including malaria control [3–5].

Globally, over 500 million school-age children are at risk of malaria infection, of these 200 million are in the sub-Saharan African region [6]. During malaria peak seasons, prevalence surveys have reported between 60 and 71% of children being infected with *Plasmodium falciparum* and 32.4% being anaemic [7, 8]. Although research has shown that school-age children suffer a disproportionate burden of malaria morbidity and mortality compared to other age groups, they are also less likely to use available malaria interventions [7, 9].

Within the context of malaria control in schools, delivery of curative and preventive health interventions, including malaria testing and treatment [10, 11] and long-lasting insecticidal net (LLIN) distributions [12–14] largely depends on primary school teachers. While studies have demonstrated the feasibility [15], effectiveness [16] and cost-effectiveness [17] of different school-based malaria interventions, others have raised concern about the acceptability and opportunity costs of engaging primary school teachers in SBH programmes. In Kenya, many teachers felt that delivering a malaria control programme was disruptive and beyond the scope of their regular work [18, 19], while in Malawi, although teachers welcome SBH programmes [10], other stakeholders have expressed reservations due to the high demand SBH services may place on teachers [5].

The sub-Saharan Africa region has one of the lowest teacher-learner ratios [20], underscoring the fact that school resources, particularly teachers, are limited. Costing of a school-based programme of malaria diagnosis and treatment has shown that when considering indirect costs alone, the cost of teachers’ time accounts for 35% and is the largest driver of annual programme costs [5], suggesting that implementing teacher-centred SBH interventions may have unintended consequences, including the potential to draw teachers and financial resources away from education-related activities to the detriment of learners’ education outcomes [21]. Therefore, ascertaining how school health interventions influence teachers’ time has relevance for SBH programmes. It however remains unclear how school health interventions influence teachers’ time as no quantitative data exist that would support or refute qualitative postulations that SBH may be disruptive [19]. In view of emerging inter-agency partnerships around school health [20] and diseases such as COVID-19 that highlight the importance of integrating water, sanitation and hygiene through schools [22], developing such an understanding, from rigorously conducted quantitative studies, will provide additional school health policy and programming insights.

In this study, the impact was evaluated of a Learner Treatment Kit (LTK) on primary school teachers’ time. LTK is a tried and tested SBH approach that includes testing and treatment of school-age children for uncomplicated malaria by teachers [5, 10]. The primary objectives of this study were two-fold: (1) to quantify primary school teachers’ time before and after the introduction of LTK; and, (2) to describe the allocation of primary school teachers’ time to different types of teaching and non-teaching activities before and after LTK.

## Methods

### Study settings

This study was conducted in Machinga, a district in the southern part of Malawi with a population of 788,256. Malaria is a disease of public health importance in the district: up to 96,000 clinically diagnosed malaria cases were treated in public health facilities in 2019 [23]. Similar to the rest of the country, transmission of malaria is stable throughout the year with a peak in the rainy season (November to March) and more than 95% of malaria infections in the district are caused by *Plasmodium falciparum* [24].

This study was conducted at 10 primary schools in Machinga district. Machinga has 183 primary schools catering for a total of 211,000 learners [25, 26]. The 10 primary schools were selected because they were all scheduled to implement a Learner Treatment Kit (LTK) during the study period. The LTK is a package of first-aid services for managing basic health ailments, such as minor injuries, diarrhoea, skin infections and uncomplicated malaria. These services are provided by teachers [10]. Although LTK services were initially designed to be available to primary school children during break times, in practice, they are provided any time based on need during school hours.

The LTK is a joint initiative between the National Malaria Control Programme within the Ministry of Health and the School Health and Nutrition Programme within the Ministry of Education Science and Technology. Save the Children provides logistical support including teacher training, supervision, procurement and distribution of supplies [27]. To qualify as LTK dispensers for school-based malaria diagnosis and treatment, teachers are trained for seven days, followed by a three-day mentorship period by qualified laboratory technicians.

### Study design

The study design used was time and motion in which continuous direct observations were performed in real time by independent observers [28]. A before and after approach was used to assess the impact of LTK on teacher’s time. Teachers trained in LTK services in the 10 study schools were observed twice: at baseline in October 2019 before initiation of the LTK activities and at endline in November 2020, 12 months after the roll-out of the LTK intervention when the primary schools were judged to be in a steady state of routine LTK service provision.

### Study eligibility

Primary school teachers who had successfully completed an LTK dispenser training were invited to participate. Trainee teachers and those who were not trained LTK dispensers were precluded from the study given they would not be allowed to test or dispense malaria treatment to learners.

Primary school teachers who agreed to participate by giving consent were observed twice, before and after the implementation of the LTK. Once recruited at baseline, we aimed to observe the same teachers at endline. To minimize the Hawthorn effect, or the change in behaviour in those observed due to awareness they were being observed [29], each teacher was observed twice during each period. The first observation session was a simulation, meant to allow the teachers to get used to being observed and to maintain their normal behaviour. The second session was for actual data collection: only data from the second sessions were used for data analyses.

The observers followed primary school teachers during their entire day at school from the time they reported for work in the morning (official reporting time is 07:30 h) to the time they stopped work at the end of the day, and directly timed teaching and non-teaching activities. During the simulation sessions, teachers were instructed to explain to the learners that the teachers were the subject of a study, otherwise the observer’s role was passive, involving no communications with teachers or learners.

### Observer training

Nine research assistants served as observers, none was a teacher. They had two days classroom-based training in time and motion studies led by an experienced observer (JC). During the training, the observers studied the main activities and their corresponding sub-activities. They were instructed (by an experienced teacher) to identify the start and end of each sub-activity without the need to ask the observed/teacher what they were doing. In addition, the observers were also instructed on how to collect data using a tablet computer. The classroom-based training was followed by a one-day pilot session in which the observers practiced observations on non-study primary school teachers. During the pilot, the observers could and were encouraged to ask questions to the observed or instructors for clarifications. Lessons learnt from the pilot were used to further modify and improve definitions and grouping of activities and the data collection tool.

### Main activities, sub-activities and analysis groups

A pre-defined tool composed of a set of activities logically organized to facilitate data collection and analysis was used to document teachers’ activities (Appendix). The tool had 8 main activities: (1) lesson plan preparation; (2) classroom teaching; (3) student supervision; (4) exercise/examination marking; (5) other teaching duties; (6) LTK; (7) administration; and, (8) non-teaching tasks. Each of the main activities had a corresponding unique list of sub-activities (Appendix). Because the LTK period coincided with the COVID-19 era, the data collection tool was modified to include a COVID-19 main activity group at endline. The sub-activities were structured in such a way that they could easily be visually identified when started and ended without the need for explanation by teachers of what they were doing.

For analysis, the sub-activities were further collapsed into five groups: direct teaching; indirect teaching; LTK services; administration; and, non-teaching tasks. Direct teaching included sub-activities such as classroom-based teaching and other teacher-learner direct supervision or coaching involving curriculum content. Indirect teaching was made of sub-activities such as making lesson notes, reading and other teaching preparation, exercise and examination markings, and supervision of learners engaged in physical exercises or sports. Administration consisted of duties such as general staff meetings, parent consultations and report writing. LTK was made up of all tasks related to providing healthcare to learners, including malaria case management such as history taking, testing and provision of anti-malarial treatment to learners. The non-teaching group was composed of informal activities including involvement in personal matters and non-work-related conversations with colleagues or other individuals. COVID-19 tasks included setting up hand-washing stations, enforcing hand washing, social distancing and wearing of face-masks, and delivery of COVID-19-related health talks to learners. To explore specific teachers’ time related to COVID-19, these tasks were treated as part of indirect teaching and as a separate group.

### Data collection

**Fig. 1:**
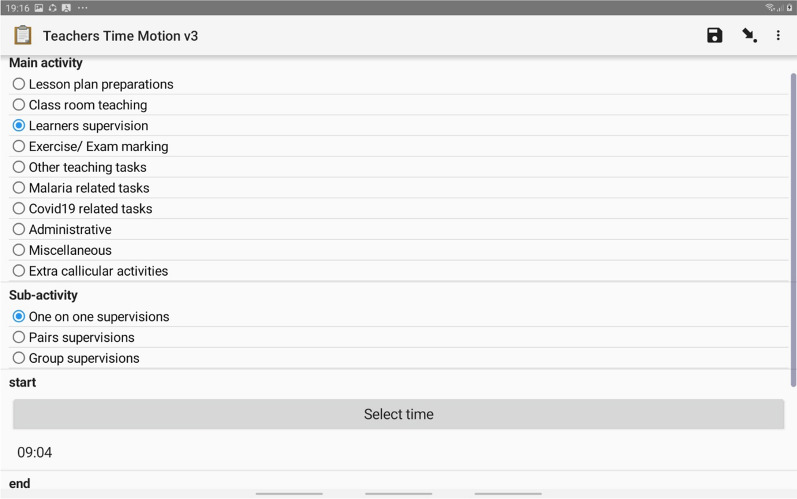
A screen shot of the time and motion data entry form. The form lists main activities and associated sub-activities which drop down once a main activity is selected

Data were collected using a structured form (Appendix), programmed digitally with Open Data Kit (ODK) software and administered using Samsung Galaxy-Tab-2.0 tablet computers. To log a sub-activity, the observer had to first identify the main activity the sub-activity was itemized under, tap on the button next to the main activity for the corresponding sub-activity list to drop down, and then tap on the button next the sub-activity of interest (Fig. 1). To start timing a sub-activity, the observer tapped on a start button and ended timing the activity by tapping on the stop button. The internal clock of the tablet computer automatically timed the sub-activities to minutes precision between tapping of the start and stop buttons. Each sub-activity was numbered serially, and the tablet automatically captured the information and date of collection.

The computer tablets were programmed to capture only one sub-activity at a time. In the rare event where teachers were performing more than one task simultaneously, for example marking an examination paper while talking to a colleague, it was up to the observer to decide which sub-activity was the dominant one to be timed. For some sub-activities, except for classroom-based teaching, teachers often switched between tasks or sub-activities; for instance, making lesson plans, chatting with a colleague, back to making lesson plans, then chatting on WhatsApp. The tool was flexible enough for the observer to accurately capture data from such sequence of sub-activities. The data were backed up in the computer tablets using SD cards and then transmitted to a central server every evening for quality control and safe storage.

### Data analysis

The primary outcome of interest was the average time in hours spent by a teacher providing direct teaching during an average day after LTK was implemented. Secondary outcomes of interest were time in hours spent providing indirect teaching, carrying out administrative duties and doing non-teaching activities per teacher. For each outcome, the time spent was estimated by adding up all sub-activity durations in minutes under respective analysis group (direct teaching, indirect teaching, administration, LTK service provision, non-teaching tasks) during the observation period for each teacher and then dividing the total by 60.

Descriptive statistics such as mean estimates for each analysis group and associated 95% confidence intervals were provided. Paired samples t-test were used to ascertain mean differences between groups before and after LTK. Statistical significance was defined by a *p* value < 0.05. Each outcome whose mean significantly changed before and after LTK was further used as a dependent variable in a repeated measures mixed regression model, to allow for the correlation between observations contributed by each teacher in both the pre- and post- intervention periods [30]. The empirical regression models took the form:

$${\text{Y}}_{{{\text{it}}}} = \beta_{0} + \beta_{{1}} {\text{LTK}}_{{\text{t}}} + {\text{X}}_{{{\text{it}}}} \beta_{{2}} + {\text{Z}}_{{\text{i}}} \beta_{{3}} + {}_{{\text{i}}} + \varepsilon_{{{\text{it}}}}$$

where Y_it_ denotes the dependent variable of interest—time in hours—for every unit (teacher observed), t is a binary variable indicating time point, taking value 0 before and 1 after LTK implementation. The main independent variable in the model is LTK, an indicator variable coded 1 if the observation was during LTK implementation and 0 otherwise. Xi and Zi are vectors of independent variables included as controls for the observed teachers (age, gender and teaching experience in years) and for the study primary schools (number of functional classrooms, number of teachers and number of teachers trained in LTK), respectively. The choice of the school and individual-level confounders was informed by the existing literature on determinants of teacher performance or motivation [31, 32]. Finally, ɑ_1_ stands for unobserved individual specific effect while ɛ_it_ represents unobserved random error term, that is normally distributed with a mean = 0.

The estimable parameters of interest are β_1,_ the effect of LTK on teachers’ time (and the main target of inference), β_2_ and β_3_ representing vectors of coefficients for individual and school level independent variables outlined above. Given the small number of teachers observed, the results were bootstrapped based on clustering at teacher level to estimate parameter standard errors and corresponding confidence intervals [33].

### Ethical approval

The College of Medicine Review and Ethics Committee (COMREC) reviewed and granted permission for the conduct of the study (P.11/18/2543). Verbal consent was sought from school headteachers and all primary school teachers before the observations started.

## Results

### Study school characteristics

The features of the 10 study primary schools are shown in Table 1. At baseline, the average number of learners and teachers per school were 1,371 and 22, respectively. The teacher-learner ratio was low before compared to the period after LTK, 1:65 vs. 1:56 while the functional classroom-learner ratio was higher before compared with the period after LTK, 1:109 vs. 1:138. Just over half (52%) of the primary school teachers had been trained on LTK. More attributes of the study primary schools are shown in Table 1.

**Table 1:** Primary school characteristics before and after LTK, N = 10

Attribute	Before LTK	After LTK
Learner population (mean)	1371	1278
Number of teachers (mean)	22	23
Percent of teachers trained in LTK	52	41
Teacher/Learner ratio	1–65	1–56
Number of functional classrooms (mean)	13	9
Classroom/Learner ratio	1–109	1–138

*LTK* Learner Treatment Kit

Seventy-four primary school teachers were observed at baseline before initiation of the LTK. Of these teachers, 64 (86.4%) were also subsequently observed post-LTK implementation. Ten teachers could not be observed during follow up because they were either transferred or on leave. These were excluded from further analysis. At baseline, the average age of the primary school teachers was 34.7 years, 32.3% were female and mean years of teaching experience was 8.7 years (Table 2).

**Table 2:** Primary school teacher characteristics at baseline, N = 74

Attribute	Mean	Min	Max
Age in years	34.7	24	53
Percent female	32.3	21.8	44.4*
Years of teaching experience	8.7	1	27

*95% confidence interval

### Duration of primary school teacher observations

In total, 739.8 h of teacher observation took place. More hours of observation took place before LTK: 430.0, range 0.1–7.9 h compared with after LTK period: 309.8 h, range 2.5–8.4. The average time spent observing a teacher was greater at baseline relative to after LTK: 5.8 vs. 4.8 h, p = 0.01.

### Proportion of primary school teacher time before and after LTK

The proportions of time primary school teachers devoted to direct and indirect teaching were 40.1 and 17.2% before LTK compared with 43.2 and 18.1% after LTK. The cumulative percentage of time teachers committed to core teaching activities (teaching and administrative duties) was approximately 76% and did not change substantially before and after the introduction of LTK in the primary schools. Teachers spent 2.9% of their time on LTK services and this was largely at the expense of non-teaching activities as shown in Table 3.

**Table 3:** Distribution of primary school teacher time by activity, before and after LTK

	Before LTK		After LTK	
Main activity	%	Cumulative %	%	Cumulative %
Direct teaching	40.1	40.1	43.2	43.2
Indirect teaching	17.2	57.3	18.8	62.0
Administration	18.4	75.7	15.3	77.3
Non-teaching	24.3	100.0	19.8	97.1
LTK			2.9	100.0
Total	100.0		100.0	

*LTK* Learner Treatment Kit

### Primary school teacher time (in hours) before and after LTK

In absolute terms, primary school teachers on average spent more hours per day at school 5.81 h, 95% CI (5.13–6.21) before LTK compared with 4.84 h, 95% CI (4.49–5.19) after LTK was introduced, and this difference is statistically significant. The amount of time teachers devoted to teaching (both direct and indirect) remained the same before and after LTK, but there is evidence teachers spent less time on administrative duties (0.74 h, 95% CI: 0.68–0.83 *vs* 1.07 h 95% CI: 0.85–1.21) and on non-teaching activities (0.96 h 95% CI: 0.76–1.14 vs. 1.41 h 95% CI: 1.22 to 1.78) during LTK period compared with the period before LTK*.* Primary school teachers spent on average 0.14 h (range: 0.05–0.96) per day providing LTK services to learners as shown in Table 4.

**Table 4:** Primary school teacher time (in hours) before and after LTK according to main activities

	Overall		Before LTK		After LTK		p-value^b^
	Mean	95% CI	Mean	95% CI	Mean	95% CI	
Direct teaching	2.22	2.03–2.41	2.33	2.05–2.61	2.09	1.84–2.33	0.21
Indirect teaching	0.96	0.86–1.06	1.00	0.85–1.15	0.91	0.77–1.04	0.35
Administration	0.90	0.79–1.02	1.07	0.85–1.21	0.74	0.68–0.83	0.01
LTK					0.14	0.05–0.96^a^	NA
Non-teaching	1.24	1.04–1.45	1.41	1.22–1.78	0.96	0.76–1.14	0.02
Total	5.30	4.96–5.64	5.81	5.13–6.21	4.84	4.49–5.19	0.01

*LTK* Learners Tool Kit, *95% CI* 95% Confidence interval. ^a^Range, *NA* Not applicable, ^b^P values by paired samples t-tests

### Effects of LTK on teacher administration and non-teaching time

Primary school teachers’ participation in LTK is associated with a substantial reduction in the average time devoted towards administrative duties. An increase in the mean number of classes per school decreases whereas an increase in number of LTK-trained teachers increases the number of hours the average teacher spends on administrative duties as shown in Table 5, model A. Similarly, participation in LTK is associated with a substantial reduction in the amount of time teachers spend on non-teaching activities. While increasing age, number of classes per school and being a female teacher led to a reduction in time spent on non-teaching tasks, increasing number of teachers per school increased the time engaged in non-teaching activities (shown in Table 5, model B).

**Table 5:** Effects of LTK on teacher administration and non-teaching time, adjusted for individual and school level controls

Dependent variable Administration time		Model A				Dependent variable Non-teaching time		Model B			
Covariates	Coef.	Bootstrap		95% CI		Covariates	Coef.	Bootstrap		95% CI	
		Std. Err.	P value					Std. Err.	P value		
LTK	− 0.162	0.082	0.040	− 0.323	− 0.011	LTK	− 0.584	0.207	0.005	− 0.990	− 0.178
Age	− 0.005	0.011	0.681	− 0.027	0.017	Age	− 0.069	0.031	0.027	− 0.131	− 0.008
Gender	0.052	0.098	0.597	− 0.140	0.243	Gender	− 0.548	0.230	0.017	− 0.999	− 0.097
Years of teaching	0.010	0.013	0.439	− 0.015	0.035	Years of teaching	0.035	0.033	0.282	− 0.029	0.099
Number of classes	− 0.030	0.011	0.010	− 0.053	− 0.018	Number of classes	− 0.181	0.032	< 0.001	− 0.244	− 0.119
Number of teachers	0.015	0.005	0.309	− 0.005	0.016	Number of teachers	0.058	0.015	< 0.001	0.028	0.088
Number LTK trained	0.057	0.023	0.012	0.012	0.101	Number LTK trained	0.024	0.049	0.624	− 0.073	0.121
Constant	0.201	0.355	0.571	− 0.494	0.896	Constant	4.264	0.831	< 0.001	2.635	5.894

*LTK* Learners Treatment Kit, *Coef* coefficient, *Std. Err* Standard Error, *CI* Confidence Interval

### COVID-19 and teacher time

**Fig. 2:**
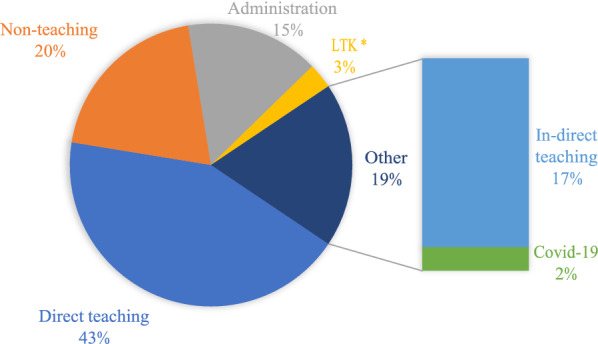
Percentage distribution of teacher time when COVID-19 tasks are treated as part of indirect teaching and as a separate group. *Learner Tool Kit

The post-LTK period coincided with COVID-19 era, in which changes to mitigate or reduce the risk of COVID-19 transmission were put in place. These changes included reducing the number of hours teachers/learners spent in schools per day. The study explored how COVID-19-related tasks influenced teacher time. As shown by Fig. 2, COVID-19-related tasks accounted for 2% of overall teacher time (in absolute terms this is 0.10 h, range: 0–0.38 h) and took place at the expense of indirect teaching activities.

## Discussion

There has been concern that school health programmes may be at a cost to time for teaching [21]. In this study, test of this hypothesis found that a school health programme (LTK) did not substantially affect core teaching activities. This was because there is spare capacity, as up to 24.3% of primary school teachers’ time is spent on informal non-teaching activities. The average amount of time teachers spend on informal activities was substantially lower after LTK compared with the period before LTK, suggesting that primary school teachers responded to additional health-related tasks by reallocating their time from informal activities to more essential health service provision. This is in contrast to how teachers reacted to the introduction of health activities in schools in another setting (China) in which there was a possible overall reallocation of school resources from academic inputs to health inputs [21]. The programming and policy implications of these findings in this setting are that tasking primary school teachers with SBH programmes may not have huge opportunity costs and that teachers should continue playing central roles in the delivery of SBH programmes.

This is the first time and motion study within the context of SBH programmes in a low-income country. There are no relevant previous studies against which the results may be directly compared. Nonetheless, there are three key findings from this study. First, the proportion of time primary school teachers devote to providing SBH care to learners is small, approximately 3%, which equates to a teacher spending an average 0.14 h (range: 0.05–0.96) per day on health-related services. In contrast to prevailing perceptions [5], these results suggest that primary school teachers can be tasked to provide SBH without undue concern that such services will exert excess burden on their workload. Second, the cumulative percentage of time teachers devote towards core teaching activities (teaching and administrative duties) is approximately 76% and did not change substantially after the introduction of LTK in primary schools. The introduction of LTK, at least in the short term, does not therefore appear to be detrimental to core teaching services. Others have reported similar results [5]. It can be argued that LTK as currently implemented can be introduced in primary schools as an integral part of school health interventions to promote learner health but its impact on core teaching functions need to be monitored long term. Third, the average amount of time teachers spent on informal/ non-teaching activities was substantially lower after LTK (0.96 h, 95% CI: 0.76–1.14) compared with the period before LTK (1.46 h, 95% CI: 1.22–1.78), suggesting the substitution of informal with formal activities in the face of additional SBH-related workload in schools. This short-term adaptation by primary school teachers should be monitored long term, given that evidence of long-term behaviour would be indicative of both programme sustainability and minimal negative consequences on core teaching functions. Finally, an important result is that the mean time teachers spent in school was greater before relative to after LTK: 5.8 vs. 4.8 h. This is probably not due to the intervention (LTK) but to the measures put in place to reduce the transmission of COVID-19. Such measures included tasking teachers to work in shifts to reduce potential risk of exposure by shortening duration of time teachers spent in school per day.

This study provides intuitive results as regards some determinants of teachers’ engagement in informal or non-teaching activities. The model predicts that increasing the number of classes per school reduces the amount of informal activities for each teacher, probably reflecting lack of spare time in view of high workload that comes with extra classes, while increasing number of teachers per school increases the time teachers spend on informal activities. In the same vein, an increase in the number of teachers trained on LTK increases time teachers spend on administrative duties, which may reflect the additional specific administrative and reporting requirements imposed by the LTK intervention.

It was noted that current studies primarily focus on analyses of short term health and education benefits of SBH programmes [2, 5]. Further studies should ascertain broader social benefits of SBH in schools, i.e., benefits beyond health and education gains for learners or impact on teachers.

The first relates to estimations of household healthcare cost savings accruing from health expenditure reductions due to averted malaria complications, as a result of prompt uncomplicated malaria treatment in school-age children by teachers, and care-related productivity gains, i.e., saving of caretakers’ productive time resulting from averted need for complications care and convalescence. These cost savings have potential micro-level economic consequences as studies have shown that economic costs of inpatient malaria treatment in this age group can be substantial [34]. Additionally, in settings where there is little formal health insurance coverage [35], households tend to deploy informal coping mechanism to minimize health shocks in children, including reducing non-medical consumptions [36]. Thus, the broader benefits of averted school-age children illnesses due to malaria complications is likely to be significant, underscoring that savings from averted complications have the potential to increase household disposable income leading to increased consumption, with potential to enhance welfare for both school-age children and the household. These benefits should be quantified and be part of the evidence base to inform school health policies and strategies in the region. As others have noted, failure to recognize full economic value of health interventions can result in policy makers and programmers unwittingly undervaluing health interventions, which translates into under investments and sub-optimal policies [37]. Such future studies should also appraise long-term socio-economic benefits in school health interventions with a gender perspective. SBH has disproportionally high effect on girls, which can have enormous policy implications.

## Strengths and limitations

The strength of the study is premised on use of time and motion method, generally regarded as the most reliable means to quantify time utilization and ascertaining how time is allocated to different types of activities, compared to alternative approaches such as work sampling and time efficiency studies [28].

This study has some limitations. First, while the before and after study design used has the temporal advantage of being being able to suggest that the outcomes of interest (amount of time teachers devote to teaching activities) are influenced by the intervention, this design does not have control over unobservable or omitted variables that may also be changing at the same time the intervention is implemented [38]. Therefore, it is challenging to fully attribute observed changes in outcomes to the intervention. In this study, to control for omitted variables, as many covariates were included as were available from the dataset. However, it is plausible that other potential covariates were not included, which may have biased the results. Second, it is acknowledged that some amount of teacher activity (e.g. exercise markings and lesson plan preparations) may have taken place at teachers’ homes during non-official hours. As the period of observations was limited to official times when teachers were in school premises, not taking off-site teaching activities into account can result in biasing estimates downwards. Although there is no reason to assume that off-site activities differed in a significant way before and after LTK implementation, future qualitative studies are proposed which can provide explanatory depth and should assess any changes in teachers’ off-site behaviour within the context of school health interventions assessments. Specifically, such inquiries should ascertain if teachers adapt to added health-related workload by shifting some teaching tasks to home, which otherwise would be done during formal times in schools. Third, the sample was restricted to rural schools only. Therefore, generalizing the findings to primary school teachers of all schools (urban and rural) is not possible. Nevertheless, since the malaria burden in children is generally high in rural compared to urban settings [39], focusing attention on rural schools/teachers may be warranted for informing policies to tackle malaria in school-age children. Fourthly, the post-intervention period coincided with COVID-19-related mitigation efforts, including reduced teacher and learner schedules implemented by the Ministry of Education to reduce COVID-19 transmission. Disentangling the effects of such changes from the effects of LTK on teacher time is challenging. As the time teachers spent specifically on COVID-19-related tasks was small (2%), COVID-19 changes appear to have had minimal effects on how teachers use and allocate their time overall. Finally, this study did not test whether there were any detrimental effects of LTK on learning, which could have been done using standardized tests. This would assess whether LTK had effects on quality of teaching in addition to quantity of teaching as reported in this study.

## Conclusions

This study demonstrates that introduction of a school health programme (LTK) in primary schools is not detrimental to core teaching activities. Primary school teachers can manage their time to ensure that the additional time required for SBH services does not come at the expense of teaching or other work-related duties. The programming and policy implications of these findings are that tasking primary school teachers with SBH programmes does not have substantial opportunity costs, therefore, teachers should continue playing a key role in the delivery of SBH programmes in the region to promote learners’ health.

## Data Availability

The datasets used and/or analyzed during the current study are available from the corresponding author on reasonable request.

## Appendix

See Table 6

**Table 6:** Structured form used to collect data showing main activities, sub-activities and analysis groups

	School Name			
	School ID			
	Teacher ID			
	Date			
	Observer ID			
	Supervisor ID			
	Main Activities		Sub-activities	Analysis groups
1	Lesson plan preparations	1.1	Reading lesson notes	Indirect teaching
	Lesson plan preparations	1.2	Writing lesson notes	Indirect teaching
	Lesson plan preparations	1.3	Collecting teaching aids	Indirect teaching
	Lesson plan preparations	1.4	Preparing teaching materials	Indirect teaching
2	Class room teaching	2.1	Class room-based teaching	Direct teaching
	Class room teaching	2.2	Class room-based instructions	Direct teaching
3	Student supervision	3.1	One on one supervision	Direct teaching
	Student supervision	3.2	Group supervisions	Direct teaching
4	Exercise/ Exam marking	4.1	Marking of class works	Indirect teaching
	Exercise/ Exam marking	4.2	Marking of examinations	Indirect teaching
5	Other teaching tasks	5.1	Physical exercise classes	Indirect teaching
	Other teaching tasks	5.2	Sports	Indirect teaching
	Other teaching tasks	5.3	Hand craft etc	Indirect teaching
6	LTK related tasks	6.1	History taking	LTK
	LTK related tasks	6.2	Malaria testing	LTK
	LTK related tasks	6.3	Malaria treatment	LTK
	LTK related tasks	6.4	Recording test/ treatment	LTK
	LTK related tasks	6.5	Counselling about fever	LTK
	LTK related tasks	6.6	Any other medical care	LTK
7	Administrative	7.1	General duties—e.g. meeting parents	Administration
	Administrative	7.2	Archiving materials/ records	Administration
	Administrative	7.3	Supervising cleaning of school’s ground	Administration
	Administrative	7.4	Arrives at school premise	Administration
	Administrative	7.5	Departs school premises	Administration
	Administrative	7.6	Other (specify)	Administration
8	Non-teaching	8.1	Chatting with colleagues	Non-teaching
	Non-teaching	8.2	Eating	Non-teaching
	Non-teaching	8.3	SMS/ WhatsApp texting	Non-teaching
	Non-teaching	8.4	Personal calls	Non-teaching
	Non-teaching	8.5	Staying idle	Non-teaching
	Non-teaching	8.6	Walking about aimlessness	Non-teaching
	Non-teaching	8.7	Other (Specify)	Non-teaching
9	COVID-19 tasks	9.1	Giving COVID-19 health talks	COVID-19
	COVID-19 tasks	9.2	Setting up hand washing utensils	COVID-19
	COVID-19 tasks	9.3	Enforcing socials distancing	COVID-19
	COVID-19 tasks	9.4	Enforcing wearing of face masks	COVID-19
	COVID-19 tasks	9.5	Any other COVID-19 related tasks	COVID-19

## Abbreviations

COMREC: College of Medicine Research and Ethics Committee
COVID-19: Corona virus diseases 19
LTK: Leaner tool kit
ODK: Open data kit
SBH: School based health
